# Alcohol Use and Depression: A Mendelian Randomization Study From China

**DOI:** 10.3389/fgene.2020.585351

**Published:** 2020-10-19

**Authors:** Chen Zhu, Qihui Chen, Wei Si, Yingxiang Li, Gang Chen, Qiran Zhao

**Affiliations:** ^1^College of Economics and Management, China Agricultural University, Beijing, China; ^2^Beijing Food Safety Policy and Strategy Research Base, Beijing, China; ^3^China Center for Genoeconomic Studies (CCGS), Beijing, China; ^4^WeGene, Shenzhen, China; ^5^Hunan Provincial Key Lab on Bioinformatics, School of Computer Science and Engineering, Central South University, Changsha, China

**Keywords:** alcohol use, depression, Mendelian randomization, genetic instruments, *ALDH2* rs671, *ADH1B* rs1229984, China, alcohol consumption

## Abstract

**Background:** Alcohol use has been linked to a number of physical conditions, but the relationship between alcohol drinking and depression, one of the most common mental disorders that is a significant contributor to the global burden of disease, is still under debate. We aim to help fill the literature gap on the causal effect of alcohol use on depression by using genetic instruments of *ALDH2* rs671 and *ADH1B* rs1229984 in the Mendelian randomization (MR) framework.

**Materials and Methods:** We collected a sample of 476 middle-aged and older adults from mainland China. The 10-item Center for Epidemiologic Studies Depression Scale (CESD-10) was used to measure the status of depression. The frequency and intensity of alcohol consumption were measured by (1) a binary indicator of drinking or not, (2) the total number of drinking occasions during the past 30 days, and (3) the weekly ethanol consumption in grams.

**Results:** MR estimates indicated that alcohol use was causally associated with a lower risk of depression. Parameter estimates of drinking or not (*b* = −0.127, *p* = 0.048), number of drinking occasions (*b* = −0.012, *p* = 0.040), and weekly ethanol consumption (*b* = −0.001, *p* = 0.039) were all negative and statistically significant. The results were robust after adjustments for potential confounders (e.g., income, smoking, and parental drinking behaviors), and the exclusion of heavy or former drinkers.

**Conclusions:** This is one of the first study to investigate the causal relationship between alcohol use and mental health using an MR design in East Asian populations. Further studies are needed to clarify the mechanisms of this causal link.

## Introduction

Alcohol use has been linked to a large number of physical conditions, and recent work has challenged the conventional view that low-to-moderate alcohol consumption has a beneficial health effect on coronary artery disease and diabetes (GBD 2016 Alcohol Collaborators, 2018). However, there is much less research about the causal relationship between alcohol consumption and mental health. One common but serious mental disorder, in particular, is depression, which is a significant contributor to the global burden of disease and affects one in 15 people in any given year (American Psychiatric Association, 2013). Earlier observational studies have found that alcohol use was associated with several psychological benefits (Baum-Baicker, 1985; Peele and Brodsky, 2000; Marchand et al., 2003). Whereas other studies have reported an overall null effect of moderate drinking on depression in different populations (Paschall et al., 2005; Almeida et al., 2014; Polimanti et al., 2019) and even a positive association between alcohol use disorders and major depression (Boden and Fergusson, 2011). These inconsistent findings have raised debate about the causal link between alcohol use and depression. Since alcohol use is *not* randomly assigned, one major threat of the previous observational study designs is the endogeneity issue originated from unobserved confounders and/or reverse causality. This study aims to help fill the literature gap on the causal relationship between alcohol use and depression by using genetic variants of *ALDH2* rs671 and *ADH1B* rs1229984 to instrument for alcohol use in the Mendelian randomization (MR) framework.

MR is a causal research design that uses genetic variants as instrumental variables (IV; DiPrete et al., 2018). The genetic basis of the MR approach relies on the random allocation of genes at meiosis in humans, resembling the random assignment into treatment groups in randomized controlled trials (RCT) that may be infeasible or unethical in this setting (Yeung et al., 2012; Holmes et al., 2014). We collected a sample of 476 middle-aged and older adults from mainland China, with demographics, socioeconomic status (SES), drinking behaviors, and mental health conditions linked to individual genotyping data. China is an interesting and important country in which to study this research question for several reasons: first, China has the highest alcohol-related deaths in the world (GBD 2016 Alcohol Collaborators, 2018), but studies on the causal links between alcohol use and mental health outcomes among Chinese populations are still very limited (Yeung et al., 2012). Second, depression has become a significant public health concern in China. It was estimated that the disability-adjusted life years (DALYs) of depression in China had increased by 36.5% from 1990 to 2017 (Ren et al., 2020). But little is known about the link between depression and alcohol drinking among Chinese populations. Third, both the spending and consumption of alcohol in China are still increasing rapidly. The per capita alcohol consumption in China went up from 1.7 L in 1980 to 5.7 L in 2016 (OECD, 2019) and is projected to jump to more than 10 L by 2030, exceeding the United States per capita consumption.^1^ Fourth, the proposed genetic instruments, *ALDH2* rs671 and *ADH1B* rs1229984, have a strong association with alcohol consumption and dependence, but are only prevalent in East Asian populations, which makes the MR research design more applicable in this region.

To strengthen the MR framework, we tested the instrument validity of *ALDH2* rs671 and *ADH1B* rs1229984 and dealt with threats from the pleiotropy effect, dynastic effect, and population stratification by controlling for a number of potential confounders that have not been included in most previous MR studies (e.g., parental drinking behaviors and individual genetic ancestral compositions). Besides, to avoid biased results due to inadequate separation of alcohol use levels, such as the sick-quitter bias (Paschall et al., 2005), we separated *former drinkers* from *never drinkers* and *heavy drinkers* from *moderate drinkers*.

## Materials and Methods

### Sample Collection

The survey was designed and implemented by the China Center for Genoeconomic Studies (CCGS) at China Agricultural University in the summer of 2019. The Institutional Review Board of China Agricultural University approved the protocol. Prior to data collection, all participants signed an informed consent form after receiving a careful explanation about the purpose of this study. All participants were informed that their responses were completely voluntary and confidential and were invited to contact the research team later if they had any further questions regarding any aspect of the study. Fifty villages from seven provinces in mainland China (Heilongjiang, Henan, Zhejiang, Yunnan, Xinjiang, Shandong, and Anhui) were selected; in each village, 10 households were then randomly selected. The survey collected information on participants’ regular demographic/socioeconomic status as well as detailed information about their alcohol consumption. We also collected participants’ parental drinking behaviors (i.e., father drinking or not and mother drinking or not). Additionally, 1 ml saliva samples were collected from all participants during the face-to-face interview. Excluding individuals who did not pass the quality control yielded, a total of 476 observations were collected. As reported in Table 1, the average respondent in our sample was 49.4 years old, completed 8.2 years of education, and earned CNY 70,232 (1 US Dollar ≈ CNY 7) annually.

**Table 1:** Demographic, socioeconomic and genetic characteristics of participants according to their groups of alcohol use (*N* = 476).

	Pooled *N* = 476 (100%)	Groups by alcohol use			
		Never drinkers *N* = 184 (38.7%)	Former drinkers *N* = 76 (16.0%)	Moderate drinkers *N* = 165 (34.7%)	Heavy drinkers *N* = 51 (10.7%)
	% or mean (SD)	% or mean (SD)	% or mean (SD)	% or mean (SD)	% or mean (SD)
Age	49.4 (11.6)	50.0 (11.4)	51.5 (13.6)	47.7 (10.6)	50.0 (12.3)
**Gender**					
Male	74.0%	48.7%	86.6%	89.0%	100.0%
Female	26.0%	51.3%	13.4%	11.0%	0.0%
Drinking times during the past 30 days	4.7 (6.4)	0.0 (0.0)	0.0 (0.0)	8.8 (5.8)	14.9 (2.3)
Weekly ethanol consumption (g)	62.2 (113.7)	0.0 (0.0)	0.0 (0.0)	71.6 (55.0)	347.9 (96.0)
**Depression or not**					
Yes	9.0%	16.7%	10.4%	2.1%	2.2%
No	91.0%	83.3%	89.6%	97.9%	97.8%
Years of schooling	8.2 (3.5)	7.8 (3.9)	7.9 (3.4)	8.8 (3.1)	8.8 (2.9)
Annual earnings (in 10,000 CNY)	7.0 (10.8)	6.1 (8.8)	6.7 (8.2)	8.2 (13.9)	6.7 (9.1)
**Smoking or not**					
Yes	38.3%	18.5%	40.3%	52.1%	62.2%
No	61.7%	81.5%	59.7%	47.9%	37.8%
**No. of parents that drink**					
0	19.8%	26.5%	22.4%	12.3%	15.6%
1	65.0%	59.3%	59.7%	72.6%	68.9%
2	15.2%	14.2%	17.9%	15.1%	15.5%
***ALDH2* rs671 (no. of effect alleles)**					
AA (2):	4.5%	11.1%	1.5%	0.0%	0.0%
AG (1):	31.0%	41.4%	46.3%	17.8%	13.3%
GG (0):	64.5%	47.5%	52.2%	82.2%	86.7%
***ADH1B* rs1229984 (no. of effect alleles)**					
AA (2):	42.4%	47.5%	40.3%	39.0%	37.8%
AG (1):	44.8%	39.5%	41.8%	52.1%	44.4%
GG (0):	12.9%	13.0%	17.9%	8.9%	17.8%
**Ancestral composition**					
Northern Han	49.9%	48.9%	43.7%	53.1%	52.7%
Southern Han	19.0%	21.2%	21.3%	16.9%	14.5%
Mongolian	10.0%	8.3%	11.9%	10.4%	12.1%
Japanese	2.5%	2.5%	2.2%	2.8%	2.2%
**Province**					
Heilongjiang	94	34	10	35	15
Henan	22	8	5	7	2
Zhejiang	41	18	6	12	5
Yunnan	182	78	33	54	17
Xinjiang	52	13	11	26	2
Shandong	16	7	1	6	2
Anhui	69	26	10	25	8

### Genotyping

DNA was extracted from saliva samples using the Illumina WeGene V2 Array. Imputation and quality control were performed using PLINK (1.90 Beta), SHAPEIT (v2.17), and IMPUTE2 (v2.3.1).

### Measures of Alcohol Use

We surveyed respondents about three complementary measures of the frequency and intensity of alcohol consumption. First, we asked for a binary measure of drinking-or-not status, where 0 and 1 represent *current non-drinkers* (54.5%) and *current drinkers* (45.5%), respectively. Second, we asked participants the total number of occasions that they consumed any alcohol during the past 30 days (mean = 4.7, SD = 6.4). Third, by combining the information on drinking frequency and the average amount that a participant drank on one occasion, we calculated the weekly ethanol (i.e., pure alcohol) consumption in grams as a continuous measure of alcohol use (mean = 62.2, SD = 113.7). While self-reported data often raise concerns of misreporting, it has been demonstrated that self-reported recent alcohol consumption suffers less from misreporting when multiple closed-ended questions are used and can be reliable measures of alcohol consumption (Lintonen et al., 2004). To avoid biases elevated from the inadequate separation of alcohol use levels (Paschall et al., 2005), we also classified participants into four distinct alcohol use groups of *never drinkers* (38.5%), *former drinkers* (16.0%), *heavy drinkers* (10.7%; defined as having more than 210 g of ethanol per week; Yeung et al., 2012), and *moderate drinkers* (34.8%; defined as *current drinkers* who that have less than or equal to 210 g of ethanol per week).

### Genetic Instruments

There are two genetic variants commonly used in MR studies of alcohol use: the alcohol dehydrogenase 1B gene (*ADH1B* rs1229984) and the aldehyde dehydrogenase 2 gene (*ALDH2* rs671), both of which encode enzymes involved in the metabolic pathway for ethanol and can change the metabolic balance of acetaldehyde in human body (Peng and Yin, 2009). In the human body, ethanol is first converted to acetaldehyde by alcohol dehydrogenase (ADH) and then to acetate by aldehyde dehydrogenase (ALDH).

The enzyme activity of ADH and ALDH are largely determined by the number of effect alleles (i.e., A-allele) in both *ADH1B* rs1229984 and *ALDH2* rs671. In East Asian populations, *ALDH2* rs671 alleles exist with three genotypes, GG (# of A allele = 0), AG (# of A allele = 1), and AA (# of A allele = 2), where the presence of A allele can significantly decrease the detoxification of acetaldehyde generated during alcohol metabolism in humans as noted above (Peng and Yin, 2009; Edenberg and McClintick, 2018). From Table 1, 35.5% of respondents in our sample are A-allele carriers (i.e., genotypes of AA and AG). Specifically, the percentages of genotype AA and AG are 4.5 and 31.0%, respectively. In European populations, *ADH1B* rs1229984 has been used as the principal genetic instrument in MR studies of alcohol use (Holmes et al., 2014). But because the proportion of A-allele carriers is very low (around 3% in Europeans), these MR studies require much larger sample sizes.^2^ In comparison, a majority of participants are A-allele carriers of the *ADH1B* rs1229984 in our sample (AA: 42.4% and AG: 44.8%).

### Measures of Depression

**Figure 1:**
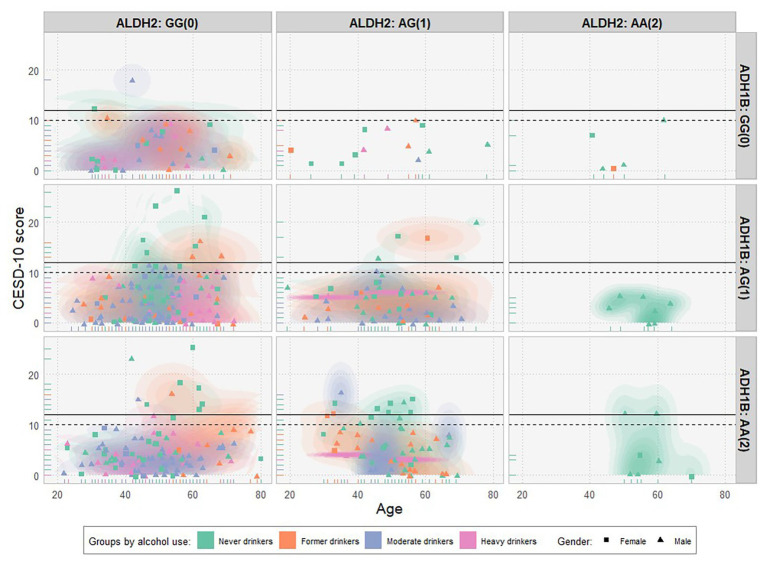
Scatter plot of CESD-10 scores over age by gender, genotypes, and groups of alcohol use (*N* = 476). CESD-10 scores over age were plotted by different combinations of *ALDH2* rs671 (horizontal) and *ADH1B* rs1229984 (vertical) genotypes (with the number of effect alleles in parenthesis). Each dot represents a single subject. The solid and dashed black horizontal lines denote for cut-off scores of 12 (optimal) and 10 for the depression, respectively. Different genders (female/male) and groups of alcohol use (never drinkers/former drinkers/moderate drinkers/heavy drinkers) were represented by distinct shapes and colors, respectively.

The 10-item Center for Epidemiologic Studies Depression Scale (CESD-10) was used as a reliable and valid survey instrument to screen for symptoms of depression (Radloff, 1977; Boey, 1999). Following Cheng and Chan (2005), we adopted the cut-off score of 12 as the optimal threshold for screening for depression. Figure 1 shows a scatter plot of CESD-10 scores over age by different combinations of *ALDH2* rs671 (horizontal) and *ADH1B* rs1229984 (vertical) genotypes. Each dot represents a single subject. Different shapes and colors demote for distinct genders (female and male) and alcohol use groups (never drinkers, former drinkers, and moderate drinkers/heavy drinkers). As expected, most of the participants with two effect alleles (AA) in *ALDH2* rs671 are never drinkers (right column).

### Statistical Analysis

**Figure 2:**
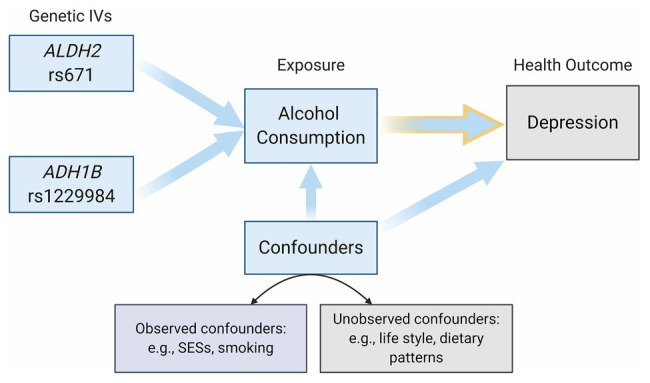
Directed acyclic graph (DAG) of the Mendelian randomization (MR) framework. This DAG illustrated the relationships between the genetic instruments (*ALDH2* rs671 and *ADH1B* rs1229984), the exposure (Alcohol Consumption), the health outcome (Depression), and the (observed or unobserved) confounders.

Multivariable linear regression was used to examine the relationship between different measures of alcohol use and depression. In the MR analyses, we first verified the validity of genetic instruments and then evaluated the causal relationship between alcohol use and depression using two-stage least squares (2SLS). Given that demographic characteristics, SES, and smoking might be highly correlated with both alcohol use and depression (Room, 2004), we adjusted age, gender, income, years of schooling, smoking, and province fixed effects in all regressions. Figure 2 illustrates the relationships between the genetic instruments (*ALDH2* rs671 and *ADH1B* rs1229984), the exposure (alcohol consumption), the health outcome (depression), and the (observed or unobserved) confounders in our MR framework. We dealt with potential threats from the pleiotropy effect, dynastic effect, and population stratification by further adjusting for parental drinking behaviors (i.e., number of parents that drink) and the individual genetic ancestral composition^3^ in MR estimations. We also performed the mediation analysis to evaluate the associations of alcohol drinking on depression explained by the years of schooling, income, and smoking. Results were reported as beta coefficients and 95% confidence intervals. All values of *p* were two-sided.

## Results

Table 2 panel *a* reports critical estimates of alcohol use on depression from separate multivariable linear regressions on all participants (column 1), the subsample excluding heavy drinkers (column 2), and the subsample excluding former drinkers (column 3). From estimates of the full sample (panel *a*, column 1), both drinking or not (*b* = −0.068, *p* = 0.022, 95% CI = −0.126 to −0.010) and the number of drinking times during the past 30 days (*b* = −0.005, *p* = 0.017, 95% CI = −0.010 to −0.001) were found to be significantly associated with a lower risk of depression, suggesting a protective effect of alcohol drinking in the prevention of depression. Exclusion of heavy alcohol drinkers (panel *a*, column 2) or former drinkers (panel *a*, column 3) did not change the estimates substantially. However, alcohol use can still be confounded by various factors even after adjustments, such as socioeconomic classes, diet patterns, physical activity, BMI, etc. Thus, the estimated associations presented in this section were not causal and needed to be interpreted with caution.

**Table 2:** Effects of alcohol use on depression – OLS and 2SLS estimation results.

	(1) All participants	(2) Excluding heavy drinkers	(3) Excluding former drinkers
	*N* = 476	*N* = 425	*N* = 400
***(a.) Multivariable linear regressions***			
**(1) Key explanatory variable: drinking or not**			
*b* (95% CI)	−0.068 (−0.126, −0.010)	−0.082 (−0.148, −0.016)	−0.065 (−0.129, −0.001)
*p*	0.022	0.015	0.046
**(2) Key explanatory variable: drinking times during the past 30 days**			
*b* (95% CI)	−0.005 (−0.010, −0.001)	−0.006 (−0.011, −0.001)	−0.006 (−0.012, −0.001)
*p*	0.017	0.012	0.019
**(3) Key explanatory variable: weekly ethanol consumption (g)**			
*b* (95% CI)	−0.000 (−0.000, 0.000)	−0.000 (−0.000, 0.000)	−0.001 (−0.001, 0.000)
*p*	0.191	0.19	0.092
***(b.) Mendelian randomization***			
**(1) Key explanatory variable: drinking or not**			
*b* (95% CI)	−0.127 (−0.253, −0.001)	−0.136 (−0.267, −0.004)	−0.149 (−0.295, −0.003)
*p*	0.048	0.043	0.045
Cragg-Donald F statistics of weak instrument tests (*p*)	44.491 (0.000)	55.917 (0.000)	34.221 (0.000)
Sargan statistics of overidentification tests (*p*)	1.837 (0.175)	1.036 (0.309)	2.061 (0.151)
**(2) Key explanatory variable: drinking times during the past 30 days**			
*b* (95% CI)	−0.012 (−0.023, −0.001)	−0.012 (−0.024, −0.001)	−0.015 (−0.030, −0.000)
*p*	0.040	0.036	0.047
Cragg-Donald F statistics of weak instrument tests (*p*)	28.664 (0.000)	36.403 (0.000)	19.967 (0.000)
Sargan statistics of overidentification tests (*p*)	1.605 (0.205)	1.595 (0.207)	2.190 (0.139)
**(3) Key explanatory variable: weekly ethanol consumption (g)**			
*b* (95% CI)	−0.001 (−0.002, −0.000)	−0.001 (−0.002, −0.000)	−0.000 (−0.001, −0.000)
*p*	0.039	0.027	0.044
Cragg-Donald F statistics of weak instrument tests (*p*)	18.113 (0.000)	20.818 (0.000)	20.386 (0.000)
Sargan statistics of overidentification tests (*p*)	1.240 (0.265)	2.559 (0.110)	2.092 (0.148)

Depression was defined as CESD-10 score ≥ 12 (Cheng and Chan, 2005). Abbreviations: 95% CI represents 95% confidence interval. All models were adjusted for age, gender, education, income, smoking, and province fixed effects. MR results were additionally adjusted for the number of drinking parents and individual genetic ancestral compositions of Northern Han, Southern Han, Mongolian, and Japanese. The First-stage Cragg-Donald F statistics (with values of p) and Sargan statistics (with values of p) are test statistics of the weak instrument and overidentification tests, respectively, which indicate that genetic instruments of ALDH2 rs671 and ADH1B rs1229984 used in MR satisfied with the relevance assumption and exclusion restriction.

The validity of using *ALDH2* rs671 and *ADH1B* rs1229984 as genetic instruments relied on the critical assumption of relevance and the exclusion restriction (Davies et al., 2018). In our research design, the instrumental relevance was satisfied with *a priori* given the robust associations previously documented (Yeung et al., 2012; Peng et al., 2019). We confirmed these correlations hold in our sample without and with the adjustments for additional controls.^4^ R-squared suggested that *ALDH2* rs671 and *ADH1B* rs1229984 together could explain 9.6–13.7% of the total phenotypic variation in different measures of alcohol consumption, indicating strong genetic instruments. We also tested for weak instruments by using Cragg-Donald F statistics in the estimation (Burgess et al., 2017; Davies et al., 2018). Another crucial concern was the potential pleiotropic effect, which occurs when a genetic IV can directly influence the outcome variable (Davies et al., 2018). There are several reasons to think pleiotropy is unlikely in our setting. First, Yeung et al. (2012) and Peng et al. (2019) considered this assumption and provided epidemiological evidence for the credibility of *ALDH2* rs671 as IV for alcohol use. Second, we consulted with PhenoScanner (v2) and found no evidence of direct links of *ALDH2* rs671 and *ADH1B* rs1229984 with depression-related phenotypes. Third, we formally conducted the overidentification test based on Sargan statistics in the estimation, which revealed no violation of the exclusion restriction, lending further support to the validity of these genetic instruments (Burgess et al., 2017).

Table 2 panel *b* reports MR results by incorporating both *ALDH2* rs671 and *ADH1B* rs1229984 as instrumental variables. The First-stage Cragg-Donald F statistics from all models exceeded the conventional cut-off of 10 for weak instruments, indicating that the two genetic IVs are jointly strong instruments in our MR design. The Sargan statistics and values of *p* of overidentification tests suggested no evidence that the genetic IVs were correlated with unobserved confounders. In all MR models, we additionally adjusted for parental drinking behaviors and individual ancestral compositions to further validate the MR settings (Willage, 2018). We found that under the MR design, alcohol use was *causally* associated with a lower risk of depression in the full sample (panel *b*, column 1). Parameter estimates of drinking or not (*b* = −0.127, *p* = 0.048, 95% CI = −0.253 to −0.001), the number of drinking times during the past 30 days (*b* = −0.012, *p* = 0.040, 95% CI = −0.023 to −0.001), and the weekly ethanol consumption (*b* = −0.001, *p* = 0.039, 95% CI = −0.002 to −0.000) were all negative and statistically significant at the 5% level.^5^ The results were robust after the exclusion of either heavy drinkers (panel *b*, column 2) or former drinkers (panel *b*, column 3). Further mediation analysis showed that the association of drinking or not with depression was mediated by approximately 11.8% through years of schooling, but not *via* income or smoking.

## Discussion

In this analysis, using a MR research design in a sample of 476 participants from mainland China, we found that the observed protective effect of alcohol use against depression was likely to be causal. The results were robust after adjustments for SES, smoking, parental drinking behaviors, genetic ancestral compositions, province fixed effects, and the exclusion of heavy or former drinkers.

This is one of the first studies to investigate the causal relationship between alcohol use and mental health using an MR design. The findings are in line with previous research that reported regular alcohol consumption was associated with better mental health conditions and lower levels of depression (Baum-Baicker, 1985; Peele and Brodsky, 2000; Marchand et al., 2003; Polimanti et al., 2019). Our study contributed further evidence that among a sample of middle-aged and older adults (with an average age of 49.4) from mainland China, alcohol use was causally associated with the prevention of depression. Paschall et al. (2005) reported no statistically significant associations between moderate alcohol use and depression, but the sample they used was young United States adults with an average age of 21.8 years old. Boden and Fergusson (2011) reported a link between alcohol use disorders and major depression based on a meta-analysis. However, they defined alcohol use disorders as a variety of alcohol misuse measures, which beyond the scope of regular alcohol use as in the current study. Almeida et al. (2014) found no significant causal effect of alcohol consumption on depression by using *ADH1B* rs1229984 as the single instrumental variable, but the analysis was based on a different population that contained 3,874 elderly (age 65–83) male participants from the metropolitan region of Perth in Australia, and the results may suffer from a weak instrument problem and lack of power since *ADH1B* rs1229984 was reported to explain only 0.24% of the variance in alcohol consumption (Rees et al., 2019).

The mechanism underlying the detected beneficial association of alcohol use and depression is still under debate. The main explanations include the psychological benefits of stress reduction and mood enhancement resulting from low to moderate drinking (Baum-Baicker, 1985; Peele and Brodsky, 2000). Hence, the role of alcohol drinking in depression and overall mental health may be a balance of the beneficial effects (likely from low to moderate drinking) and harmful effects (likely from excessive drinking; Boden and Fergusson, 2011). Among certain groups of people, such as middle-aged and older Chinese adults in our sample with limited options of entertaining and stress-relieving activities, the beneficial effects of drinking may offset the harmful effects on depression symptoms.

Before closing, we noted several caveats to our results. First, the sample used in this study was not representative of the entire Chinese population. Second, the current study was based on a sample of 476 participants, which may lack statistical power due to the small sample size, and research with a larger sample size would be preferred to confirm our findings in the future.^6^^,^^7^ Third, as noted before, the underlying mechanism of the detected beneficial impact of drinking on depression is still unclear. Further studies are needed in order to clarify the mechanisms of this causal link.

## Data Availability Statement

The datasets presented in this study can be found in online repositories. The names of the repository/repositories and accession number(s) can be found at: https://doi.org/10.6084/m9.figshare.13010567.v2.

## Ethics Statement

The studies involving human participants were reviewed and approved by the Institutional Review Board of China Agricultural University. The patients/participants provided their written informed consent to participate in this study.

## Author Contributions

CZ and QZ designed this study. CZ, YL, and GC performed research and analyzed the data. CZ, QC, and WS drafted the manuscript. All the authors read and approved the final version of the manuscript.

### Conflict of Interest

GC and YL are employees of WeGene. WeGene provided data support of collecting and analyzing genotyping data, but did not have any additional role in financially supporting the current study.

The remaining authors declare that the research was conducted in the absence of any commercial or financial relationships that could be construed as a potential conflict of interest.

## Footnotes

^1^Source: https://www.theguardian.com/society/2019/may/08/world-alcohol-consumption-on-the-rise-as-chinas-thirst-grows (Accessed: June 24, 2020).

^2^Source: https://www.ncbi.nlm.nih.gov/snp/rs1229984#frequency_tab

^3^Individual genetic ancestral composition gave the percentage of DNA that comes from different populations by comparing an individual genome to hundreds and thousands people with known ancestry and was calculated by using the ADMIXTURE program. The top four ancestries included in the MR estimation are (from high to low) Northern Han, Southern Han, Mongolian, and Japanese.

^4^Detailed first-stage estimation results are available from the corresponding author on request.

^5^Results remained robust when we used the Two-stage instrumental variables (2SIV) approach that replaced the second-stage least-squares regression in 2SLS by logistic regression (Palmer et al., 2008). Detailed estimation procedures and results are available from the corresponding author on request.

^6^Nevertheless, it should be noted that the bias resulting from small sample sizes tends to underestimate (rather than overestimate) the effect size (Dumas-Mallet et al., 2017).

^7^We replicated the results in a separate sample (*N* = 2,216) collected from China, but without available genotyping data. By using alcohol flushing reaction (i.e., a proxy to *ALDH2* rs671) as IV, we found that alcohol consumption was also related to the lower risk of depression, consistent with our main MR results. Detailed estimation procedures and results are available from the corresponding author on request.
